# Development of an adapted Clinical Global Impression scale for use in Angelman syndrome

**DOI:** 10.1186/s11689-020-09349-8

**Published:** 2021-01-04

**Authors:** Alexander Kolevzon, Pamela Ventola, Christopher J. Keary, Gali Heimer, Jeffrey L. Neul, Mathews Adera, Judith Jaeger

**Affiliations:** 1 Seaver Autism Center for Research and Treatment, Department of Psychiatry, Icahn School of Medicine at Mount Sinai, New York, NY USA; 2 Yale University Child Study Center, New Haven, CT USA; 3 Cogstate, New Haven, CT USA; 4 Angelman Syndrome Program, Massachusetts General Hospital for Children, Boston, MA USA; 5 Harvard Medical School, Boston, MA USA; 6 Pediatric Neurology Unit, Safra Children Hospital, Sheba Medical Center, Tel Hashomer and the Sackler Faculty of Medicine, Tel Aviv University, Tel Aviv, Israel; 7 Vanderbilt Kennedy Center, Vanderbilt University Medical Center, Nashville, TN USA; 8 Ovid Therapeutics, Inc, New York, NY USA; 9 CognitionMetrics, LLC, Wilmington, DE USA; 10 Department of Psychiatry and Behavioral Sciences, Albert Einstein College of Medicine, Bronx, NY USA

## Abstract

**Background:**

The Clinical Global Impression-Severity (CGI-S) and CGI-Improvement (CGI-I) scales are widely accepted tools that measure overall disease severity and change, synthesizing the clinician’s impression of the global state of an individual. Frequently employed in clinical trials for neuropsychiatric disorders, the CGI scales are typically used in conjunction with disease-specific rating scales. When no disease-specific rating scale is available, the CGI scales can be adapted to reflect the specific symptom domains that are relevant to the disorder. Angelman syndrome (AS) is a rare, clinically heterogeneous condition for which there is no disease-specific rating scale. This paper describes efforts to develop standardized, adapted CGI scales specific to AS for use in clinical trials.

**Methods:**

In order to develop adapted CGI scales specific to AS, we (1) reviewed literature and interviewed caregivers and clinicians to determine the most impactful symptoms, (2) engaged expert panels to define and operationalize the symptom domains identified, (3) developed detailed rating anchors for each domain and for global severity and improvement ratings, (4) reviewed the anchors with expert clinicians and established minimally clinically meaningful change for each symptom domain, and (5) generated mock patient vignettes to test the reliability of the resulting scales and to standardize rater training. This systematic approach to developing, validating, and training raters on a standardized, adapted CGI scale specifically for AS is described herein.

**Results:**

The resulting CGI-S/I-AS scales capture six critical domains (behavior, gross and fine motor function, expressive and receptive communication, and sleep) defined by caregivers and expert clinicians as the most challenging for patients with AS and their families.

**Conclusions:**

Rigorous training and careful calibration for clinicians will allow the CGI-S/-I-AS scales to be reliable in the context of randomized controlled trials. The CGI-S/-I-AS scales are being utilized in a Phase 3 trial of gaboxadol for the treatment of AS.

**Supplementary Information:**

The online version contains supplementary material available at 10.1186/s11689-020-09349-8.

## Introduction

### Rating scales

Neurodevelopmental disorders are associated with a wide range of symptoms that have varying degrees of severity and relative impact on the individual. Because of this heterogeneity, quantifying severity of symptoms and their change over time is uniquely challenging in these populations. The field of psychiatry faced similar challenges to those now presented by neurodevelopmental disorders, and the long history of psychometric work underpinning validated scales in psychiatry offers guideposts for developing valid and reliable clinical ratings in neurodevelopmental disorders.

Historically, in the field of psychiatry, disease-specific ratings scales have been developed to quantify symptom domain or disease-specific treatment effects. These scales are composed of individual symptom ratings (items) selected to assess the range of symptoms and signs associated with a specific diagnosis or treatment response. On many of these scales, however, items are equally weighted, and the summary scores do not adequately reflect the true condition of the individual or the importance of observed change over time.

By permitting expert clinical judgment to balance the weight and importance of individual items, adding the Clinical Global Impression-Severity (CGI-S) and CGI-Improvement (CGI-I) scales largely overcomes this shortcoming. Administered in conjunction with a comprehensive disease-specific rating scale, the CGI-S provides the clinician with a method to capture symptom severity, and the CGI-I measures change in symptoms over time in a given individual.

The CGI has gained broad acceptance since it was first developed for psychiatric applications in 1976 [1] and is routinely used in clinical trials for conditions such as schizophrenia, major depressive disorder, bipolar disorder, autism spectrum disorder (ASD), and Alzheimer’s dementia [2–7]. The validity, reliability, and sensitivity to change of the CGI, including its correlation with established disease-specific rating scales, have been assessed by a number of studies and meta-analyses [8–10]. Critically, the CGI provides reliable data only when implemented by specifically trained clinical experts in the disease under study. Furthermore, when a clinical condition lacks a disease-specific rating scale and the outcome is based on an expert overview alone, the unmodified CGI is often insufficiently sensitive [11], in part because the underlying clinical examination is not standardized.

The need for reliable clinical outcome measures has prompted the development and adaptation of the CGI for many rare conditions. These adaptations involve clear definitions of the symptom domains being rated, using descriptive anchors to support the rating of symptom levels and magnitude of change. The development of validated syndrome-specific anchors for distinct dimensions in schizophrenia (e.g., positive, negative, depressive, and cognitive) was among the first attempts to create syndrome-specific versions of the CGI scales [12]. More recently, the CGI has been adapted for rare neurodevelopmental disorders including Rett syndrome [13], Prader Willi syndrome (PWS) [14], and most recently for Angelman syndrome (AS) (NEPTUNE, NCT04106557).

### Angelman syndrome

AS was first described in 1965 and is a rare genetic neurodevelopmental condition with estimated prevalence of 1/12,000 to 1/20,000. Individuals with AS are typically diagnosed before age five and exhibit intellectual disability, speech, motor function and balance impairments, hyperexitability and various behavioral problems, sleep disorder, and seizures [15, 16]. Most cases of AS are caused by the disruption to the gene encoding ubiquitin protein ligase E3A (*UBE3A*), which can occur through a four main genetic mechanisms: whole gene deletion, UBE3A point mutation, uniparental disomy, or imprinting center defect. While individuals with AS usually exhibit developmental delay, speech impairments, movement disorder, and behavioral abnormalities, the profile and severity of clinical features varies significantly across individuals and between the genetic subtypes [17, 18]. This variation is even apparent among people with the same underlying genetic defect. For example, half of infants and young children demonstrate hypotonia, but by middle childhood and adolescence, 30% may develop hypertonia. Gait abnormalities also vary from toe-walking to stiffness or shakiness and uncontrolled jerking. Oral motor difficulties have been reported in as few as 20% and as many as 80% of individuals, depending on the study. Sleep disruption may be present in as few as 35% or as many as 72% of individuals, and sleep difficulties may peak at ages as young as 2 years or as old as 9 years [19]. This characteristic wide variability in phenotypic expression and severity presents new assessment challenges beyond those encountered in the early days of behavioral rating scale development. There is no disease-specific rating scale for AS, and creating a validated tool would be challenging due to the significant clinical heterogeneity and to the rarity of the condition. Given these circumstances, the development of disease-specific anchors for the CGI-S and CGI-I, tailored to the unique properties of AS, was undertaken to support the timely study of experimental therapeutics in AS.

The use of AS-specific anchors within the CGI to guide the clinician during the evaluation was first applied in a Phase 2 safety and tolerability study of gaboxadol for the treatment of AS (STARS, NCT02996305). This was the first time an adapted CGI-I scale was used as a pre-selected exploratory outcome measure to assess efficacy in individuals with AS. This adapted scale was developed based upon systematic literature review, caregiver interviews, and AS experts’ clinical guidance. The scale called for the independent rating of nine domains of interest identified from this process which then also formed the basis for an overall impression of severity and change. In contrast to non-AS-specific developmental assessments that were also studied in the Phase 2 trial, such as the Bayley Scales of Infant and Toddler Development [20], the adapted CGI-I scale detected significant clinical benefit of drug over placebo.

Based on the lessons learned from the Phase 2 trial, we undertook a more elaborate approach to improve the reliability and validity of the adapted scale for use in the Phase 3 trial of gaboxadol for AS [21–23]. Our own early conceptual model development work [24], broadly confirmed by that of Willgoss et al. [25], formed the conceptual basis on which the original nine domains were reduced to six broad domain categories and detailed rating anchors were developed.

## Objectives

The objective of this study was to develop and validate standardized, adapted CGI scales specific to AS for use in clinical trials in order to fill a substantial gap in the field in which no generally accepted, validated disease-specific scales exist for monitoring severity or change in AS. Aligned with the FDA guidance on Rare Disease trials [26], we sought to adapt the CGI to capture the heterogeneity of AS, as well as to demonstrate clinically meaningful treatment effects in the context of a therapeutic trial. To this end, we developed the CGI-S-AS and CGI-I-AS using AS-specific domains and clearly defined anchors within each domain based on expert clinical judgment for use in clinical trials.

## Methods

The original CGI was enhanced by developing and validating a systematic, guided disease-specific clinical interview that includes a review of the most clinically meaningful domains. Anchors for these domains, developed and refined from a series of caregiver and expert clinician evaluations, were designated to improve scale reliability and sensitivity, and the resulting numerical scales were vetted for reliability and clinical meaningfulness (Table 1).

**Table 1:**
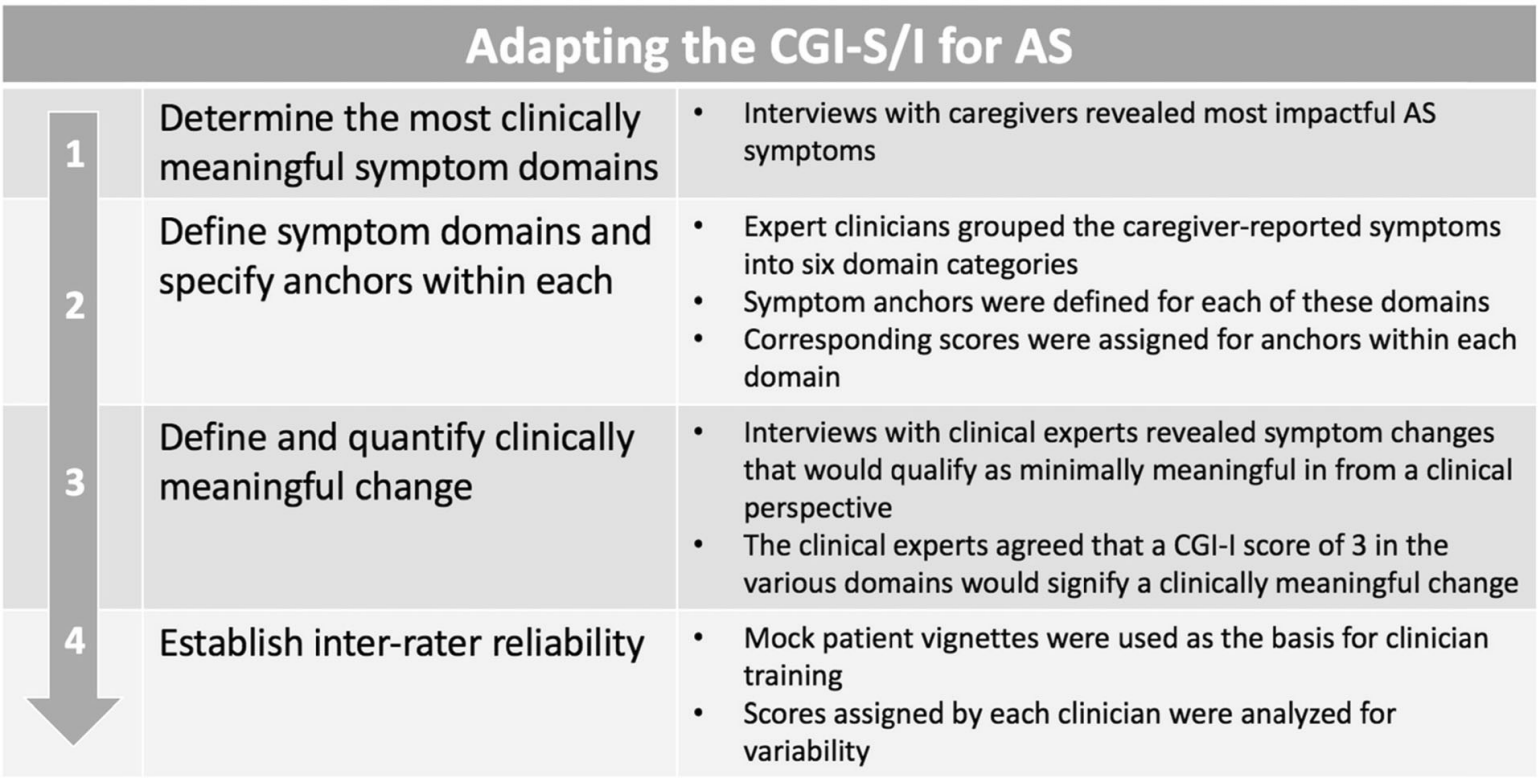
Adapting the CGI-S/I for AS

### Clinician and caregiver interviews to determine clinically meaningful symptoms

An independent market research company conducted interviews with caregivers of children with either a confirmed clinical or genetic diagnosis of AS and clinical experts. Twelve caregivers (10 mothers; two fathers) of individuals with AS aged 2–28 years, and four clinicians with expertise in AS (one psychiatrist and three clinical geneticists) participated. The caregivers were recruited from patient advocacy groups, a database held by the independent market research company, and families who had approached the sponsor as being interested in being involved in any efforts related to the work in AS. All interested caregivers were screened by the market research company, and a sample was selected. The selected caregivers did not know the pharmaceutical company sponsoring the work, and the sponsor did not know the identity of the caregivers. The clinicians were recruited from a database held by the independent market research company. The sponsor also provided names of suitable clinicians. All clinicians were screened by the market research company. As with the caregivers, the clinicians did not know the pharmaceutical company sponsoring the work, and the sponsor did not know the identity of the clinicians who were eventually included in the interviews.

The interviews were conducted one-on-one and were designed to elicit information about the most challenging features of AS. The interview questions focus on “the features of Angelman Syndrome that matter most.” (The full set of interview questions appears in Supplemental Table 1.) The questions were designed to elicit information about the most impactful symptoms of AS and factors that guide treatment decisions. The stakeholders were asked to define each of these and to indicate which are most challenging for them. The number of times each given domain was cited as being among the respondent’s top three challenges was recorded, and these domains were brought forward as most clinically meaningful.

Following these interviews to further refine the key symptom areas, a second set of interviews were conducted with 10 additional caregivers and 4 additional clinicians, recruited using the same means as above. The interviews (Supplemental Table 1) asked about quality of life impacts of AS symptoms and also elicited caregiver and clinician opinions about the relative functional importance of the key clinical features of AS (behavior, gross and fine motor function, expressive and receptive communication, and sleep). They were also asked about what meaningful improvement looks like for them.

### Expert clinicians specify symptom domains and anchors

Following the identification of these six domains (behavior, gross and fine motor function, expressive and receptive communication, and sleep), objective symptom anchors (and their corresponding severity ratings) tailored to the range of clinical presentations of individuals with AS were developed in consultation with a multidisciplinary team of clinical researchers in order to support reliable ratings between clinicians (Table 2 and 3).

**Table 2:**
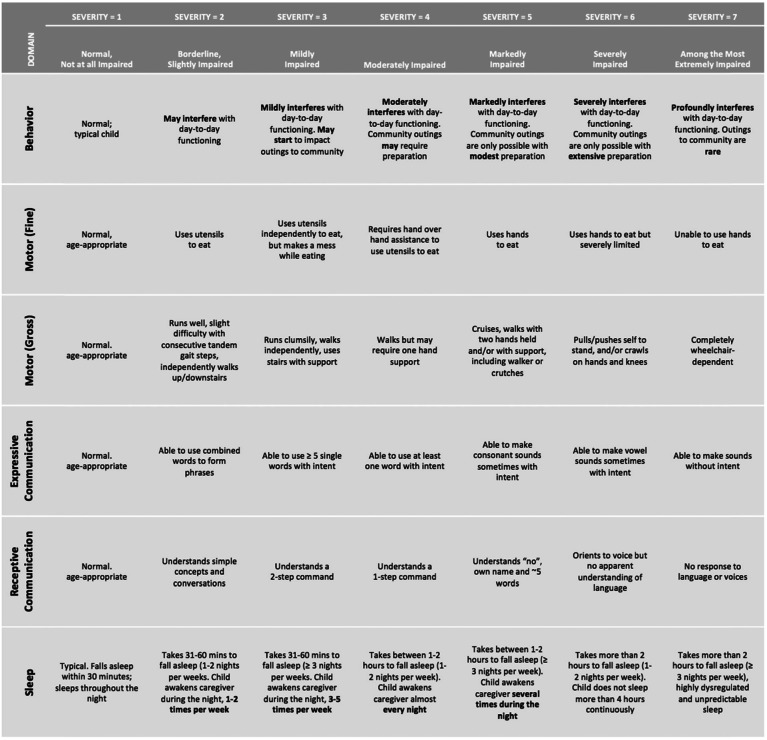
Six domains and their anchors (CGI-S-AS)

**Table 3:**
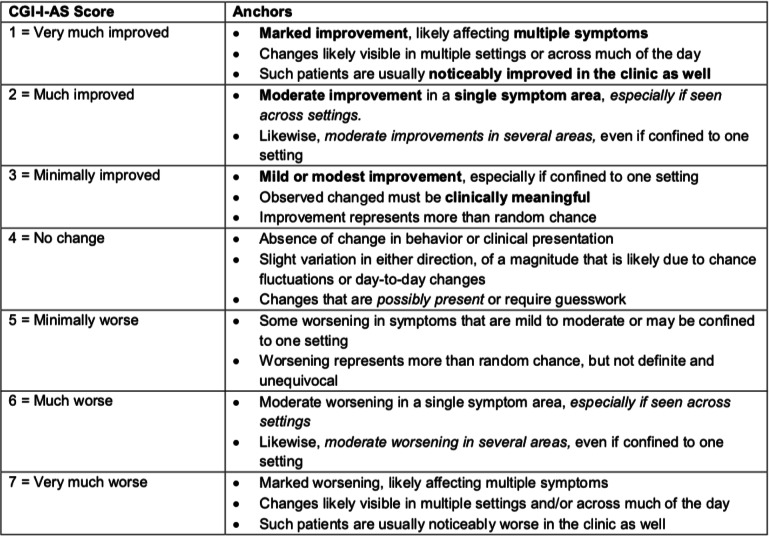
The CGI-I-AS scale

### Determination of CGI rating clinical meaningfulness

A substantial advantage to using global measures like the CGI as a clinical endpoint is that it is ideally suited for estimating and describing the clinical meaningfulness of both overall clinical severity and of change. Indeed, in situations where disease-specific rating scales made up of individual symptoms and signs are used, meaningfulness is often gauged using the CGI [27, 28].

Two different investigations were conducted to better understand the clinical meaningfulness of different CGI rating strata for each of the domains specific to AS. The first focused on both severity and change ratings and consisted of a sample of four healthcare providers and two caregiver samples, one (*N* = 5) 3-h group interview followed by individual in-person interviews for 1 h each. The second investigation focused on the expert clinician descriptions of clinical meaningfulness of change on the CGI-S/-I-AS and to examine the question of what magnitude of change would be regarded as *minimally* clinically meaningful. Nine 30-min guided interviews of clinical experts who care for patients with AS were conducted.

The first investigation of meaningfulness measured the frequency with which caregivers (parents of individuals with AS, aged 4–14) mentioned examples of behaviors within each domain as more or less challenging and their impact on QoL. The full set of interview questions appears in Supplemental Table 2. The behavioral descriptions that were provided could then be mapped on the severity anchors on the CGI-S, as could descriptions of what change might look like and the ways that change would meaningfully impact QoL. Clinicians then provided recommendations that informed mapping onto the actual CGI-S and CGI-I anchors for each domain.

### Establishment of inter-rater reliability and development of rater training materials

Two exercises were conducted to determine whether the anchors were likely to yield good inter-rater reliability in a clinical trial. In the first exercise to assess the inter-rater reliability of the severity ratings, seven clinicians with expertise in AS clinical trial methodology participated. These experts represented a range of disciplines (psychiatry, neurology, and clinical psychology) that will mirror those of the investigators who will likely rate the CGI-S/-I-AS in clinical trials. One of the clinicians interviewed two parents of children with AS, and the other six clinicians observed the video-taped interviews. An Intraclass Correlation Coefficient (ICC) was calculated to assess the inter-rater reliability of the CGI-S-AS ratings between the seven raters. Specifically, an ICC with two-way random effects, absolute agreement, and multiple raters/measurements was used (also referred to as ICC(2, *k*)). This approach was selected because the raters were from diverse disciplines and backgrounds, and the mean value of the ratings was deemed to be the most appropriate basis of the measurement [29–31].

The second exercise was intended to explore the inter-rater reliability of CGI-I-AS. Two baseline case vignettes and corresponding 12-week follow-up vignettes were developed by an expert in AS (for example, see Table 4). These written case vignettes were provided to the same seven expert clinicians. Each provided an overall improvement rating based on the 12-week case vignette (one clinician only responded to one of the case vignettes).

**Table 4:**
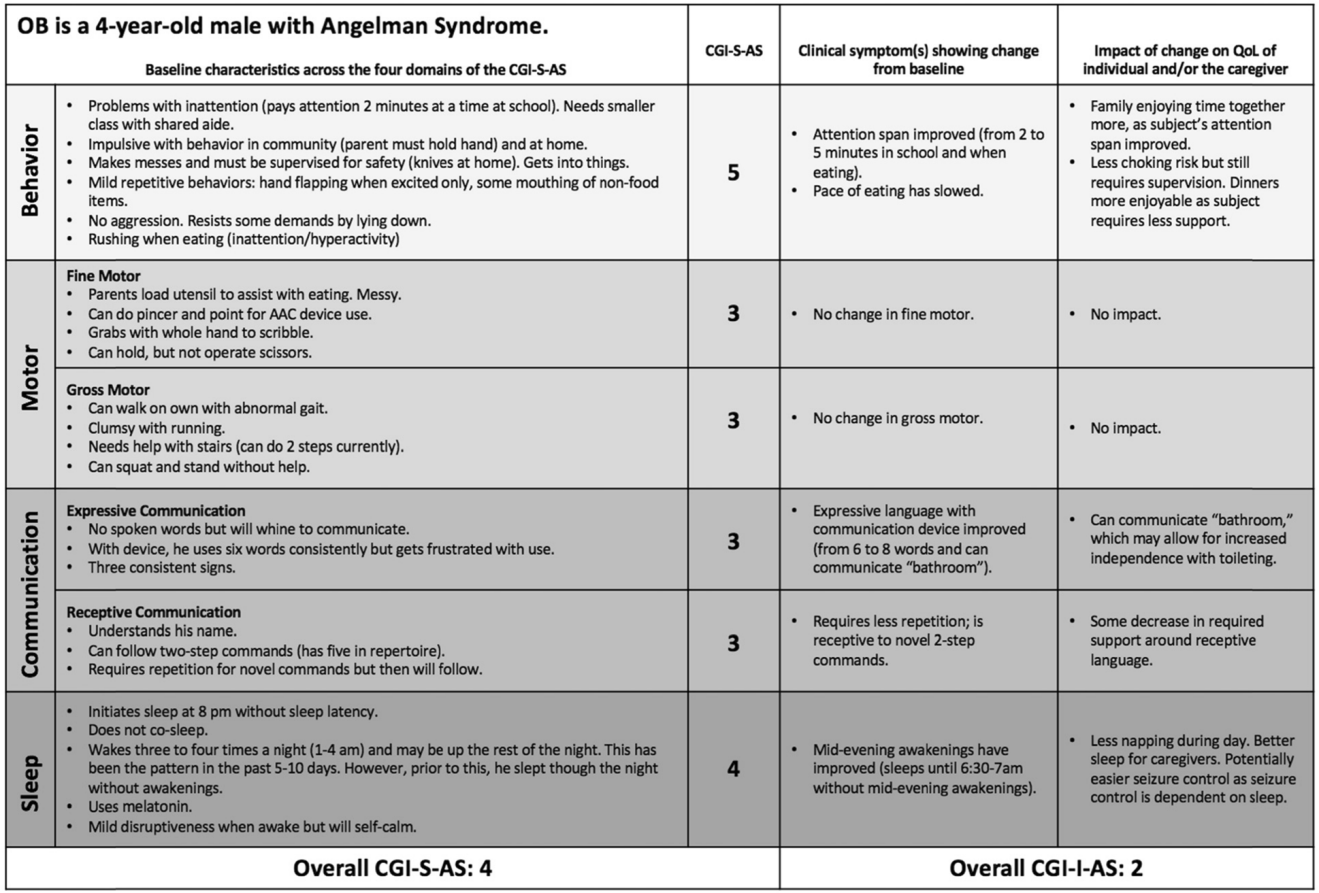
Mock patient example of CGI-S/-I-AS in use

Finally, four case vignettes of children with AS then formed the basis of an evaluation program for Phase 3 gaboxadol study raters, to be conducted after undergoing the above didactic training. A child and adolescent psychiatrist (CK) with clinical expertise in AS developed these case vignettes and then took on the role of caregiver in video-taped mock interviews conducted by another expert clinician (PV). A team of experts (comprising a child adolescent psychiatrist, clinical psychologist, and neurologist) viewed and rated these vignettes, and consensus ratings were developed on the CGI-S/-I-AS. Three of the videos were provided to all study raters before enrolling their first clinical trial participant and after they undertook the above described didactic training.

## Results

### Determination of clinically meaningful symptoms and domains

**Fig. 1:**
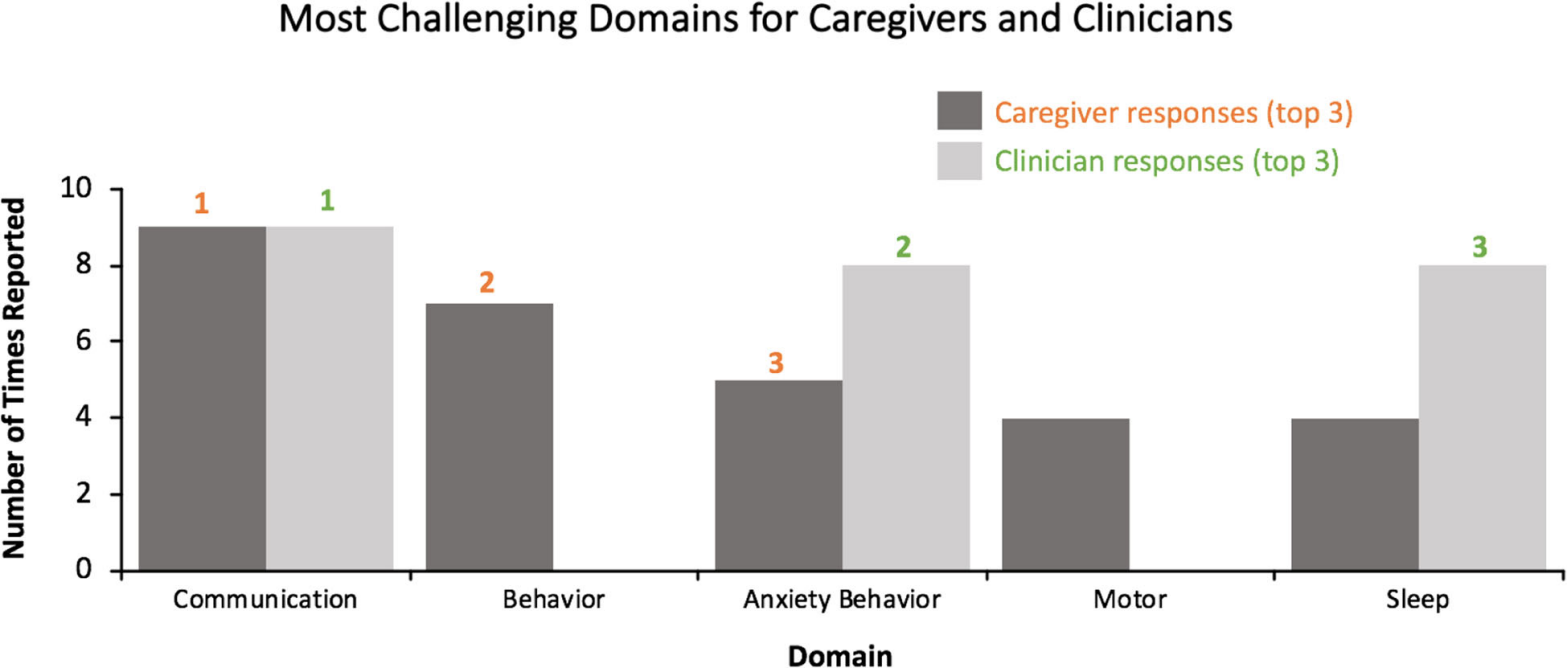
The most challenging symptom domains, as ranked by caregivers and clinicians. The number of times each given domain was cited as being among the top three challenges for caregivers (dark grey) or clinicians (light grey), and the most challenging domains ranked 1–3 (orange: caregiver rank; green: clinician rank)

Quantification of these caregiver responses revealed that communication and behavior symptoms were most commonly rated as the most challenging (Fig. 1); communication was ranked first and tied to QoL, and behavioral symptoms were ranked second, but noted as the hardest symptom domain to manage in day to day life. It is noteworthy that caregivers regarded anxiety as part of the behavioral presentation of their child with AS and that anxiety manifests in the form of behavioral disturbances. Among clinicians, behavioral symptoms were most impactful on QoL (Fig. 1). Sleep and motor functioning were also determined to present significant challenges. Caregivers’ views on the importance of sleep were divided: some indicated it was highly impactful and a major problem, whereas others (mainly caregivers who indicated they had developed effective strategies) did not indicate it was among the most problematic. Clinicians were more likely than caregivers to rate sleep as a top concern, not only for the patient but also for family QoL. Clinicians also noted the direct link between sleep and other co-occurring problems, such as behavior and non-epileptic myoclonus.

Based on results from the caregiver and clinician interviews, the final six domains for inclusion in the CGI-S/-I-AS were behavior, fine and gross motor, expressive and receptive communication, and sleep. The behavior domain encompasses a wide range of behavioral symptoms, including atypical and maladaptive behavior with a strong emphasis on how behavior impacts both the individual’s functioning and the functioning of the family. The gross motor domain assesses the ataxia common in AS and use of large muscles for locomotion/movement (e.g., standing/walking/running). The fine motor domain assesses the use of hands and fingers for functional tasks (e.g., feeding). The communication domain, divided into receptive and expressive communication, measures the ability of the individual to understand and to utilize expressive language, including non-verbal communication. The sleep domain encompasses general sleep behavior, with a focus on the specific sleep problems in AS, including sleep onset delay and multiple awakenings throughout the night.

### Specification of symptom domain anchors

Anchors were developed to define the symptom-based domains, and rating levels included a focus on symptom severity and impact on functioning (Table 2). The behavior domain includes a wide range of maladaptive behaviors seen in individuals with AS, such as aggression, hyperactivity, anxiety, and repetitive behaviors. The rating is based on how significantly the behavior interferes with functioning and the level of support required from caregivers, teachers, and clinicians. A CGI-S-AS rating of 3 (Mildly Impaired), for instance, reflects mildly interfering behavior or behavior that may impact community outings, whereas a rating of 6 (Severely Impaired) reflects severe impact on day to day functioning or community outings such that required extensive preparation is required. Of note, individuals with AS also have characteristic positive behavioral qualities not addressed in this scale as its purpose is to gauge potential treatment benefits. Adverse effects, including those potentially impacting the positive qualities, such as good mood that is often reported in AS, would be captured elsewhere. The sleep domain anchors reflect the sleep symptoms commonly seen in AS, such as delayed sleep onset, nighttime wakening, and co-sleeping with a caregiver. A rating of 3 (Mildly Impaired), for example, indicates that the child takes between 31 and 60 min to fall asleep (≥ 3 nights per week) or the child awakens the caregiver during the night, 3–5 times per week. A rating of 6 (Severely Impaired) indicates that the child takes more than 2 h to fall asleep (1–2 nights per week) or the child does not sleep more than four hours continuously. A rating of 7 indicates the child takes more than 2 h to fall asleep (≥ 3 nights per week), with highly dysregulated and unpredictable sleep.

For the domains that reflect skill development (motor; communication), the anchors were designed to align with developmental hierarchies and reflect deviations from typical development. In the gross motor domain, the anchors include pulls to stand and crawls (severity rating of 6, Severely Impaired), walks with hands held (severity rating of 5, Markedly Impaired), and runs but clumsily (severity rating of 3, Mildly Impaired). The anchors also include supports commonly used by individuals with AS (e.g., wheelchairs, walkers, or crutches). In the fine motor domain, the anchors were designed with a focus on feeding as a key example of a fine motor task. The skills range, for example, from reliant on caregiver for feeding (severity rating of 7, Among the Most Extremely Impaired) to feeding with hands (severity rating of 5 Markedly Impaired), to use of utensils (severity rating of 2, Mildly Impaired), to normal/age appropriate (severity rating of 1, Normal/Not At All Impaired). The receptive and expressive communication domains focus on core language, and the anchors were selected to reflect the typical developmental progression of these skills. Anchors for receptive communication include no response to language, corresponding to a rating of 7 (Among the Most Extremely Impaired), understands ~ 5 single words, corresponding to a rating of 5 (Markedly Impaired), and understands simple concepts and conversations, corresponding to a rating of 2 (Borderline, Slightly Impaired). Anchors for expressive communication similarly progress from able to make sounds without intent (7; Among the Most Extremely Impaired), to use of one single word with intent (4; Moderately Impaired), to use of phrase speech (2; Borderline, Slightly Impaired). Importantly, use of Augmentative and Alternative communication (AAC) devices or sign language, which is common in the AS population, is included in the ratings (Table 2).

While each CGI-S-AS domain is scored individually using specific anchors relevant to that domain, the global CGI-S-AS score does not represent an average of the domain scores but rather the overall gestalt of the patient’s condition and function as perceived by the physician. In contrast to the CGI-S-AS, individual domain ratings are not captured on the CGI-I-AS. However, a CGI-I-AS form for capturing narrative descriptions of change in each of the domains is employed to assure comprehensiveness of examination and documentation of the impact of change on the patient and family to determine its magnitude of clinical meaningfulness and to assure optimal scale reliability and validity for the global improvement rating.

### Clinical meaningfulness of CGI severity and change ratings

Transcriptions and notes from both investigations were reviewed for common themes and overlap of concepts. Results revealed that the communication domain was at the core of many of the QoL concerns raised by parents, with the most extreme consequences including inability to convey pain or unhappiness, miscommunication translating into frustration, outbursts, and mislabeling with social marginalization as a consequence. Problems such as inability to vocalize full phrases or inability to communicate beyond priority needs were rated as least impactful on QoL. Concerns with following instruction and need for reinforcement of instruction were rated as intermediate with respect to impact on QoL. However, it was in these intermediate areas where clinically meaningful changes could be envisioned and described. For example, the child’s ability to communicate with intentionality and consistency, as well as their ability to internalize and remember instructions without need for repetition or guidance, was regarded as the most meaningful. These caregivers were able to give specific examples of the types of improvements that would be meaningful, along with descriptions of how these would impact QoL. Clinician recommendations based on these observations were made for optimal training content for validity and reliability of ratings.

Not surprisingly, within the behavioral domain, physical aggression and dropping to the ground at unpredictable moments were among the most challenging aspects. Distinct AS behaviors could be ranked on magnitude of impact; for example, aggression, hyperactivity, and pica (the eating of materials that are not edible) had the greatest impact, and inappropriate laughter and motor stereotypies had the least impact on QoL. Meaningful changes in the more disruptive behaviors were readily defined by the parents and linked to specific benefits to daily life for the caregiver, patient and family including reduction of stress, improved safety of the child, and, importantly, improved social interaction, opportunity for outings, and other similar improvements.

Constraints on outings were another theme in caregiver descriptions of the burden associated with the gross motor manifestations (e.g., impaired ambulation) and fine motor manifestations (e.g., impaired ability to eat with utensils) of AS. Meaningful change was described in terms of independence of movement and reduced need for caregiver support, affording greater independence and the opportunity to utilize caregivers who may be unable to lift the child. Independence (especially in eating) was a theme for improved fine motor functioning as well; however, the greatest meaningful impact of improvement in fine motor function according to caregivers was improvement in the ability to use a touchpad assistive communication device. Improved communication was noted to lead to less frustration, fewer behavior problems, and reduced stress.

Disorders of sleep including, at their most challenging, night awakenings with disruptions to caregivers (crying, hitting a wall, roaming), as well as less disruptive aspects such as delayed sleep onset, daytime sleepiness, and bedwetting, were unsurprisingly identified as causing substantial burden. These challenges result in caregiver fatigue, marital tensions when a parent must regularly sleep with the child, inability to go out for an evening owing to difficulties finding a sitter able to provide necessary support, disruption of parental routines that bolster health (e.g., daily exercise), and vocational effectiveness. Parents anchored examples of meaningful improvements in quantitative terms (e.g., fewer disrupted nights per week) and distinguished problems with sleep onset from night awakenings in terms of their likely impact on caregivers and family.

Overall, this investigation of caregiver impressions of the magnitude and nature of differences in severity and change that are clinically meaningful fully aligned with results from the individual interviews with nine clinical experts. These experts were clinical researchers with specific expertise in AS. All were affiliated with academic medical centers or children’s hospitals. They included one clinical psychologist, one clinical geneticist, four neurologists, two children and adolescent psychiatrists, and one Master’s-level psychometrician. Six were based in the USA, two were in Europe, and one in Israel. These experts were first asked to define clinically meaningful change in the context of AS. Uniformly, all reported that clinically meaningful change in symptoms would result in improved functioning and lead to positive impact on quality of life for patients and caregivers.

Noteworthy themes emerging from qualitative analysis of interview transcripts and notes included the capacity for improvement in one domain to influence another domain. For example, an improvement in fine motor functioning can facilitate expressive communication with assistive devices. Improved expressive communication reduces frustration, which may result in reduced aggressive or maladaptive behaviors. Also almost all of the expert clinicians indicated that sleep improvements are likely to have the greatest impact on the QoL of caregivers and other family members and may further improve behavior. Behavior was described as among the most amenable to change with treatment, as compared to communication and motor functions where limitations may be more fixed in AS.

After providing answers to these open-ended questions about the overall scale and the individual domains, the experts were asked to comment on the CGI-I-AS anchors for global improvement (Table 3). Without exception, a rating of 3 was regarded as reflecting minimally clinically meaningful change. Note that the anchors for a rating of 3 on this scale include the statement that the “observed changes must be clinically meaningful.” The experts agreed that “minimal improvement” (CGI-I-AS = 3) would reflect a mild or modest improvement in at least one setting that is deemed by caregivers to be meaningful (with respect to function or quality of life), but that may not be seen by others who see the patient less frequently or may not be detected by a less sensitive tool. However, to warrant a rating of 3, improvements must represent more than random chance. Furthermore, it was noted that a change in the CGI-S would not be expected with an improvement rating (i.e. CGI-I-AS) of 3 and may not even occur with ratings of “much improved” (CGI-I-AS = 2). Given the severity of the AS underlying condition, the magnitude of change that would be detectible as a change in CGI-S-AS is much larger than is detectible using the CGI-I-AS. A CGI-I-AS rating of 2, “much improved,” would be assigned in cases where moderate improvement was evident in at least one symptom area and, especially, if seen across settings. Likewise, moderate improvements in several symptoms, even if confined to one setting, might warrant a rating of 2. Ratings of 2 would unequivocally indicate treatment response and very likely suggest continuation of treatment if available. Ratings of 1, or “very much improved,” may be rare in the context of short-term clinical trials in neurodevelopmental disorders. Ratings of 1 would indicate marked improvement, likely affecting multiple symptoms, and are likely evident across multiple settings, including observable in the clinic.

Although the CGI-I-AS was designed to capture global change, interviews with experts also elicited domain-specific descriptions of what change would qualify for ratings of “minimal,” “much,” and “very much improved.” There was a high level of agreement between experts in these descriptions which can be easily mapped to the descriptions of the parents. Experts all agreed that for all domains, a “minimal improvement” (CGI-I-AS = 3) could be modest in magnitude but must be seen consistently in at least one setting to assure a small improvement did not occur by chance. Examples in the behavior domain might include longer periods of focus on schoolwork, reduction in number, duration and/or intensity of aggressive or disruptive behaviors, reduction in interference from behaviors, or improved response to transitioning between caregivers or changes in routine. Minimal improvement in expressive communication is rated in the presence of one additional non-verbal sign or gesture to communicate needs, ability to use one useful (functional or meaningful) new word or an increase in attempts to communicate needs in a non-spontaneous manner (potentially through greater use of a communication device). In receptive communication, improved ability to respond to instructions would be rated as minimal improvement as would other manifestations of clearer understanding that were reliably observed (e.g., through increased eye contact and attention in the presence of instruction or social interaction). Experts noted that providing reliable ratings for receptive communication is more difficult than measuring change in expressive communication. In the motor domain, slight improvement in standing, stair-climbing, posture, walking, or rate of falls would be rated as minimal improvement given the functional significance of these small gains as for similar reasons would fine motor function changes seen in a single meaningful action such as holding a pencil, writing, use of cutlery, grasping, more accurate reach or interface with devices, or assisting in dressing or handwashing. Experts noted that improvement in motor functions (with the exception of signs like tremor) may be difficult to detect during the time course of a typical clinical trial and are difficult to measure in children as there will be different references for normality based on the child’s age. For sleep, “minimal improvement” would be rated if there was an improvement on at least one of several factors, including changes in sleep onset time, duration, number of awakenings, and agitation at night. Sleep is a major area of concern in AS, and even small changes can have large impact on affected individuals and their caregivers.

Relative to ratings of minimally improved, the CGI-I-AS rating of 2 (“Much improved”) in each of these domains is assigned when changes are seen across settings, with greater frequency, consistency, or effectiveness; produce improvements to function; and/or are noticed by more than one caregiver. A rating of “Very much improved” (CG-I-AS = 1) in each of the domains describes fairly dramatic improvements in magnitude and consistency, across settings and observers resulting in noticeably improved functionality and/or independence. Overall, there was agreement across clinical experts about the anchors they use and overlap in the clinical descriptions of what each of these rating levels would look like on the CGI-I-AS. These descriptions are an integral part of the rater training program.

### Inter-rater reliability and use of rater training materials

Two exercises were conducted to determine whether the raters could reliably utilize the domain anchors. Results from the first exercise, related to the CGI-S-AS, listed in Table 5, found ICC values to range from 0.847 to 0.996, indicating good to excellent reliability between raters (i) in absolute terms and (ii) when using as a reference previous studies using the same approach [32]. Since these were cross-sectional interviews of parents, this exercise did not permit examination of the inter-rater reliability of CGI-I-AS.

**Table 5:** ICC values for the six CGI domains and overall CGI-S-AS summary score

Domain	ICC (2, *k*)
Behavior	0.98288
Fine motor	0.98875
Gross motor	0.99622
Expressive language	0.96953
Receptive language	0.99538
Sleep	0.96189
CGI-S overall	0.84677

In the second exercise, ratings for both cases were highly consistent across the raters. For the first case, all CGI-I-AS ratings except one were within one point of each other (five raters coded CGI-I-AS as 2, one rater coded a 3, and one rater coded a 1), and for the second case, all ratings were within one point of each other (five raters coded CGI-I-AS as a 2 and one rater coded a 1).

A didactic training that covered the development of the CGI-S/-I-AS and an overview of the completed scale were next developed. This training described, in detail, the domains and anchors, as well as mock case examples with their corresponding CGI-S-AS and CGI-I-AS ratings. The training also covered caregiver-reported examples of clinically meaningful improvement (described in detail below) and impact on QoL for each of the domains. Lastly, potential pitfalls to the use of the CGI, such as recall bias and inter-rater variability, as well as mitigation strategies for these, were described. For example, to avert experimental error due to reporter variability, raters are encouraged to interview the same parent or caregiver of a subject throughout the trial. This helped ensure that differences in CGI-S/-I-AS scores over time were not due to differing perspectives from caregivers who might interact with the subject in different environments. Similarly, trial sites should when possible use the same trained rater for each subject throughout the trial. Raters are encouraged to take notes during the examination on observations and reports that underlie each CGI-S/-I-AS score and to review these notes in each subsequent study visit wherein the tool will be used. This is done to reduce recall bias over a multi-week trial, especially for sites with multiple subjects.

Finally, clinicians viewed and rated the case vignettes using the developed CGI-S-AS and CGI-I-AS scales. Ten of the 19 study raters met the reliability threshold, defined as ALL CGI-S-AS and CGI-I-AS domains, and global scores rated within one point of the consensus codes for the three videos. Of the nine raters who missed the reliability threshold, seven raters missed the threshold on one code and two raters missed the threshold on two codes. A fourth video was therefore provided to these nine raters, and all met the reliability criteria on this fourth video.

## Discussion

This report describes a systematic approach that was employed to develop, validate, and train raters on a standardized, adapted CGI specifically for AS. The shortage of reliable biomarkers and validated clinical outcome assessments in many other rare neurodevelopmental disorders has rendered the CGI a widely used tool in this field [2]. It was included as the pre-specified primary exploratory endpoint in a phase 2 trial of gaboxadol in AS, where preliminary success inspired further refinements to the scale and its application to support a phase 3 trial.

The resulting CGI-S/I-AS scales (Supplemental Figures 3 and 4) capture six critical domains (behavior, gross and fine motor function, expressive and receptive communication, and sleep) defined by caregivers and expert clinicians as the most challenging for patients with AS and their families. In an attempt to balance the need to address the salient signs and symptoms of AS with the need for ease of use, several behavioral symptoms were combined into a single domain; aggression, anxiety, stereotypies, and hyperactivity are all included under the behavior domain. Although this approach may result in patients with different behavioral problems scoring similarly on this domain, we suggest that the overall behavioral disturbance is still captured and is most clinically relevant in assessing severity and improvement. Additionally, the narrative capture form that was developed as part of the CGI-I-AS to record in detail the specific changes that have occurred in each domain will allow this information to be available for later examination.

Although epilepsy is a dominant feature in AS and can influence the patients’ development and function, it was not included as a domain in the CGI-AS scales because it is not specific to the developmental or behavioral profile of AS. This tool was designed to capture treatment effects on the core features of AS, rather than common comorbidities such as epilepsy, scoliosis, or constipation, each of which have their own effective treatments. Furthermore, seizures can be readily assessed and quantified by existing tools, including electroencephalography and seizure diaries (such as those used in the phase 3 development program to capture this important domain).

Another goal when adapting the CGI for AS was to address the fact that the traditional CGI-S scale is scored from 1 to 7, where “1” signifies “normal” function. Thus, in the adapted CGI-S-AS, nearly all patients would be scored at or above “3.” This raises the question of whether, as applied in AS, the CGI-S-AS is effectively a 5-point rating scale rather than a 7-point scale. However, we believe that retaining “normal function” on this scale is important because a chief application of the scale is assessment in clinical trials where potentially disease-modifying treatments such as gene therapies will eventually be tested.

Although this instrument may be of high impact and value to the field of AS, the approach had some limitations. The caregivers interviewed to determine important domains may not have fully represented all caregivers’ concerns. We attempted to address this concern by interviewing 12 different caregivers to try to capture the heterogeneity of this diagnosis. Further, the clinicians involved in the reliability analyses were expert physicians in the field of AS. These individuals are likely representative of Principle Investigators who will lead clinical trials, which is the context for which the scale was developed, but they do not necessarily represent the physicians who may use this scale more broadly within clinical practice. While our results demonstrate that the scale has the potential for excellent reliability in a clinical trial with adequate training, for effective use as a clinical tool in the hands of non-expert AS clinicians, the validity would have to be examined separately.

## Conclusions

Results from this study suggest that rigorous training and careful calibration for clinicians implementing the CGI-S/-I-AS scales will allow it to be reliable in the context of randomized controlled trials. We envision the CGI-S/-I-AS scales for use in AS clinical trials broadly and present them herein so that future studies can aim to test their use in larger samples and provide subsequent validation.

## Supplementary Information

**Additional file 1:** Supplementary materials.

## Data Availability

The datasets generated during and/or analyzed during the current study are not publicly available but could become available in an appropriately modified format from the corresponding author upon reasonable request
