# Supplementary material for: Development of an adapted Clinical Global Impression scale for use in Angelman syndrome
Source: J Neurodev Disord. 2021 Jan 4;13:3. doi: 10.1186/s11689-020-09349-8 (PMC7784030; doi:10.1186/s11689-020-09349-8)
Supplement: Supplementary file 1 — Additional file 1: Supplementary materials. [file 11689_2020_9349_MOESM1_ESM.docx]

**SUPPLEMENTAL MATERIALS**

**Supplemental Table 1. Stakeholder interview questions for determining clinically meaningful symptom domains.**

| Initial stakeholder interview questions   - What are the features that matter most to Angelman syndrome caregivers? - What are their day-to-day experiences, especially challenges and pain points? - Which aspects of Angelman syndrome do they consider to be most limiting to their child’s quality of life? - What are their support networks and influences? - What decision factors go into their child’s therapy and medication choices? |
| --- |
| Follow-up stakeholder interview questions (for further refinement)   - What are the key symptomatic challenges within the six key domains (expressive and receptive communication, behavior, sleep, fine and gross motor skills) that most impact day-to-day QoL? - What does meaningful improvement look like for families of children with AS in these domains? - For the clinicians: What are clinicians’ gut reactions to the proposed CGI scales [domains] and are there potential areas for optimization? |

**Supplemental Table 2. Stakeholder interview questions for determining clinical meaningfulness of symptom changes.**

| HCP Overview   - To get a sense of the perspective you bring, can you provide us with an overview of the area of focus in which you practice? - Over the course of your career, how many Angelman syndrome patients have you evaluated?   - Are you currently seeing any patients with Angelman syndrome?   - If so, how many patients? - Do you use the CGI scale to evaluate Angelman patients?   - If so, what are you using it for? (go to question below)   - If not, what tool or assessment are you using to assess Angelman patients?   - How does that tool measure clinically meaningful change? |
| --- |
| Defining *Clinically Meaningful Change*   - What does the term *clinically meaningful change* in Angelman syndrome mean to you? (How would you define it?) - Have you seen clinically meaningful change you just described in your AS patients? |
| Perhaps we can discuss some examples of what that looks like (or would look like):   - Think of a AS patient under your care and please describe what clinically meaningful change has looked like as it relates to:   - Behaviour   - Motor   - Communication   - Sleep - What is the smallest change that you would consider meaningful? - What would increasing scores look like? - What is the importance/impact of minimally meaningful change on the caregiver? |
| (While sharing the CGI-I-AS scale with the interviewee):   - Considering the change you just described, how would you score it on the CGI-I-AS? Why would you give this score? - What would a CGI-I-AS score of 3, 2 and 1 look like for this patient (including motor, communication, behavior, and sleep)? - How is this *minimally* meaningful change important from a caregiver perspective? What is the impact of this change? - Are improvements in one domain more valuable than another? |
| Closing   - Do you have any final feedback or thoughts? |

**Supplemental Table 3. The full CGI-S-AS Record Form.**

**
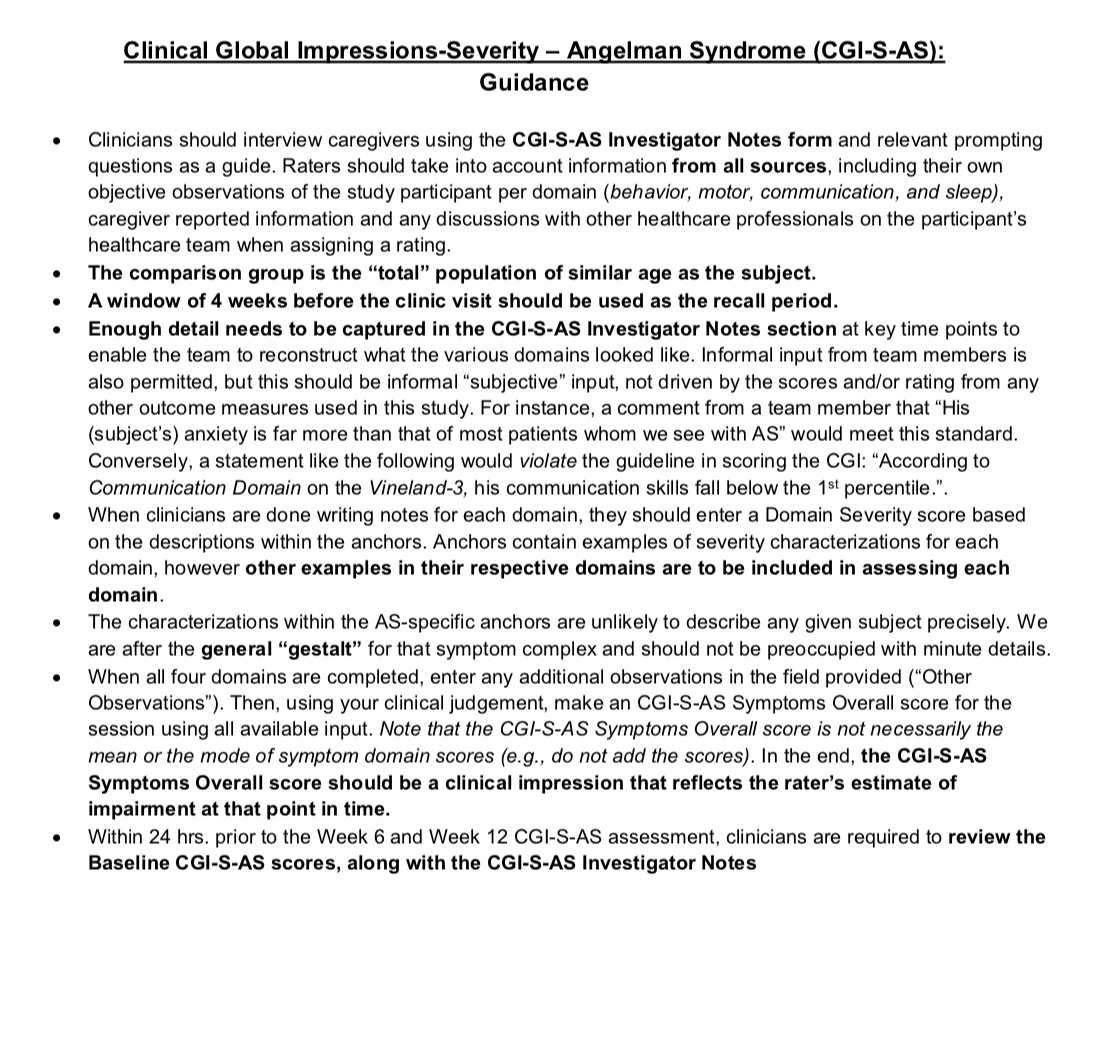
**© 2019 Ovid Therapeutics Inc. All rights reserved.

**
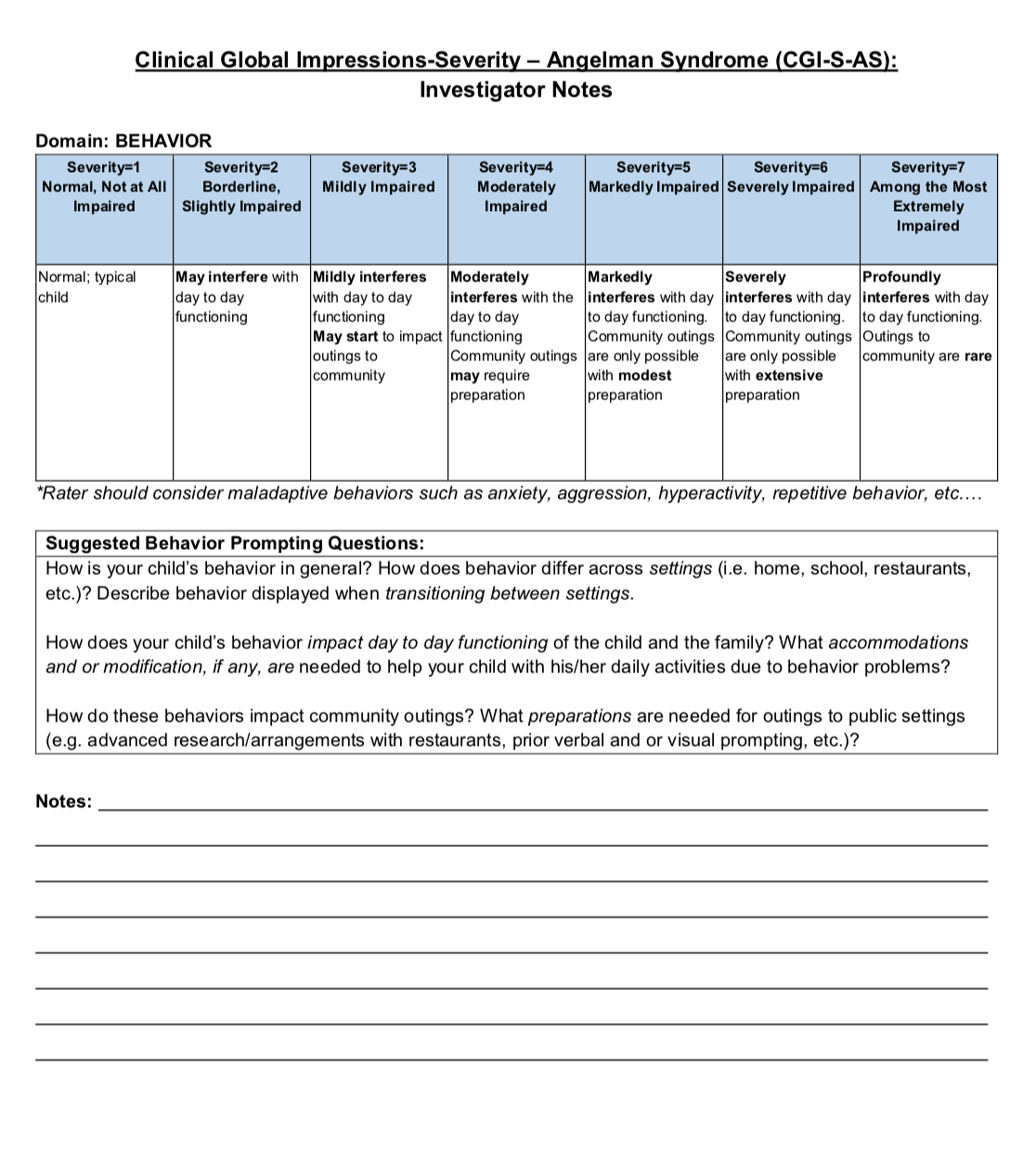

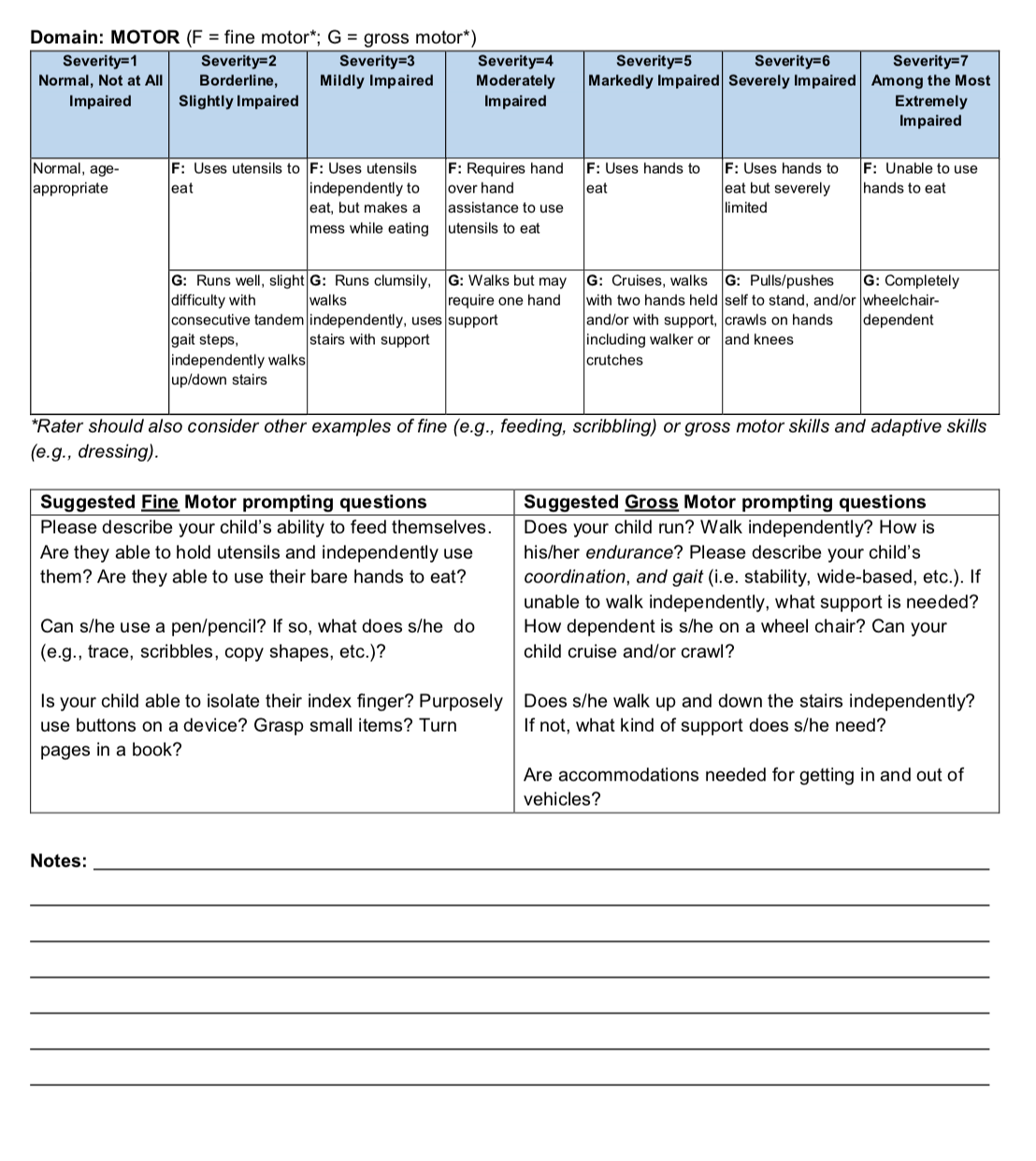

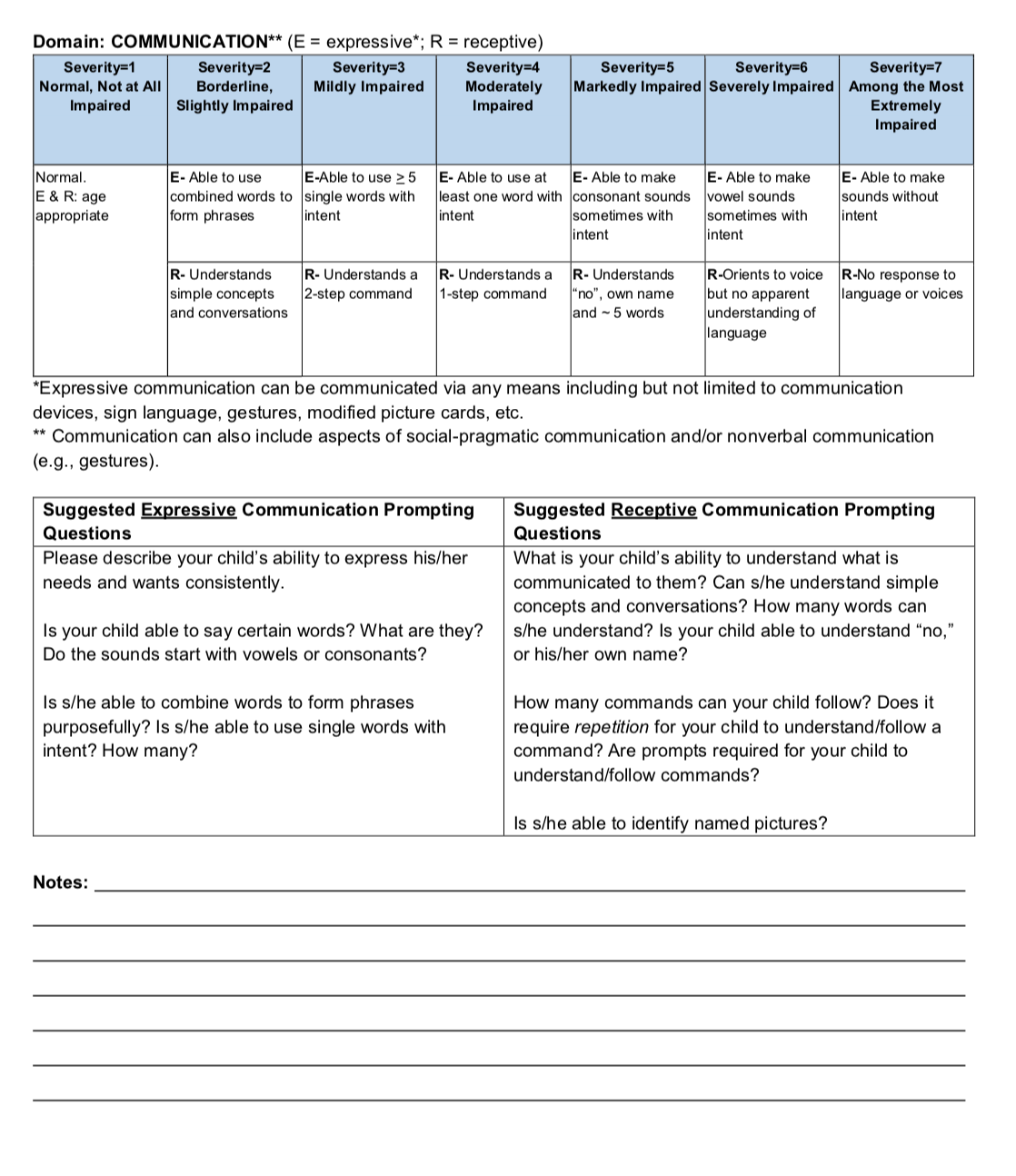

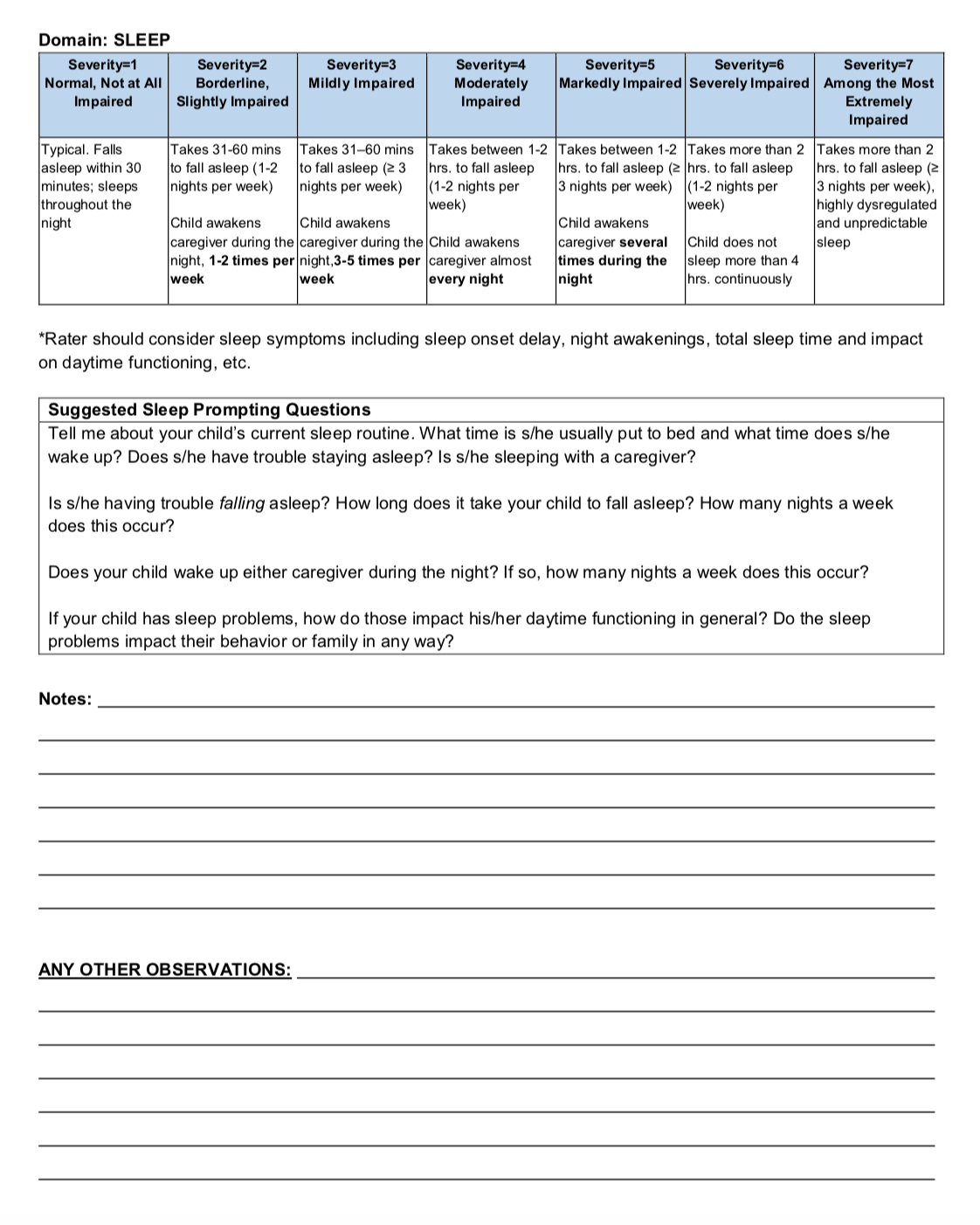

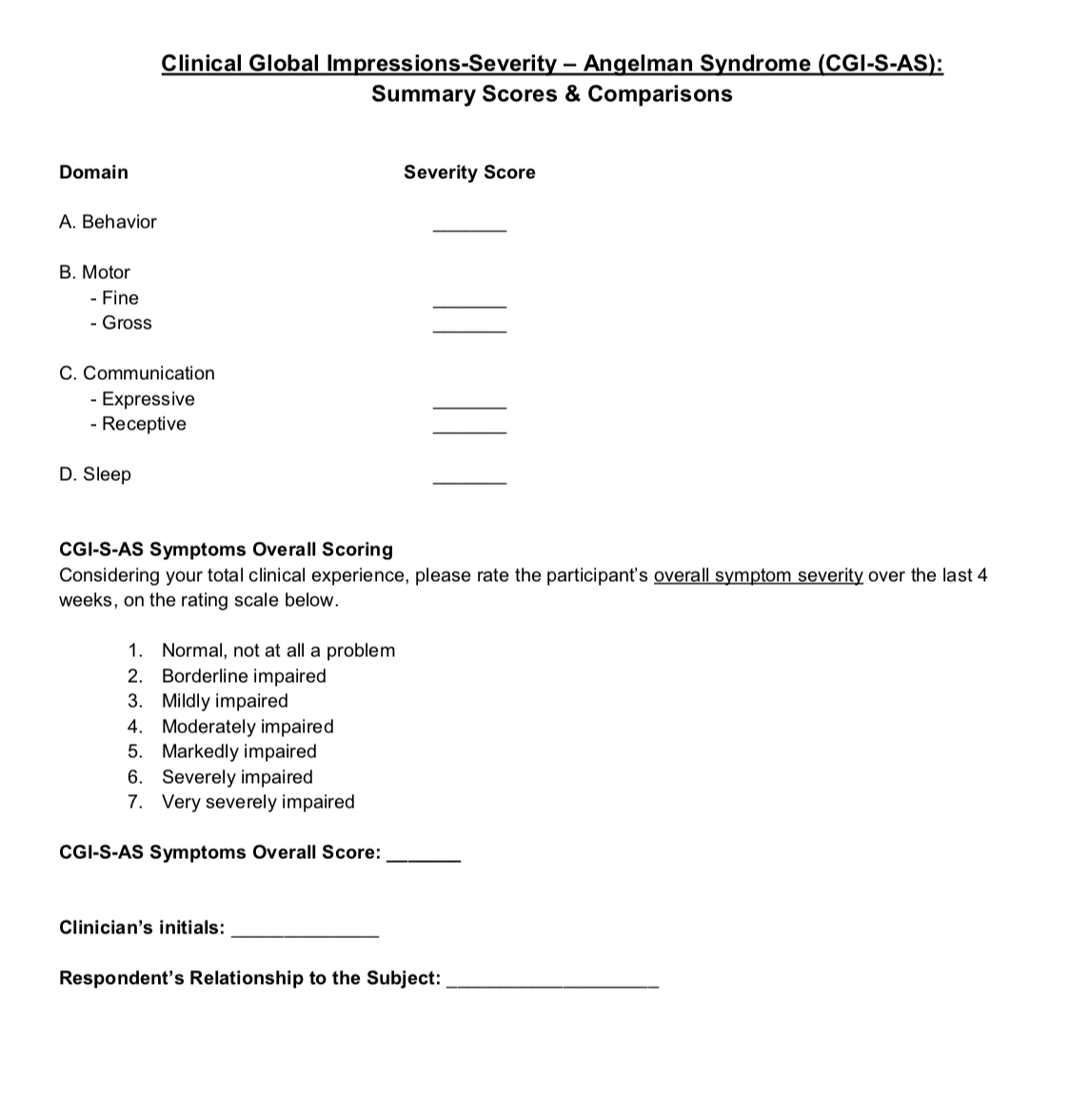
**

**Supplemental Table 4. The full CGI-I-AS Record Form.**

**
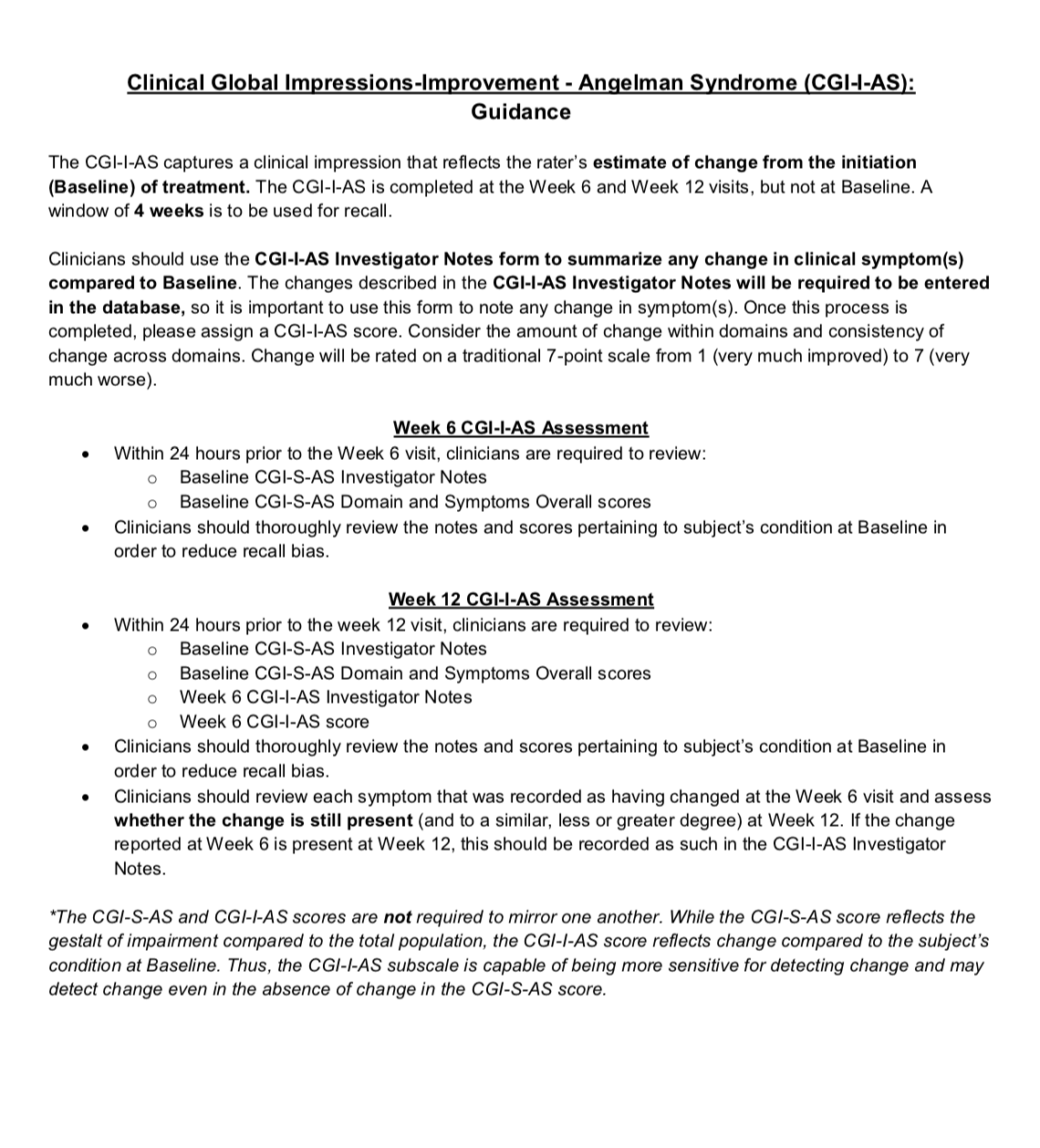
**© 2019 Ovid Therapeutics Inc. All rights reserved.

**
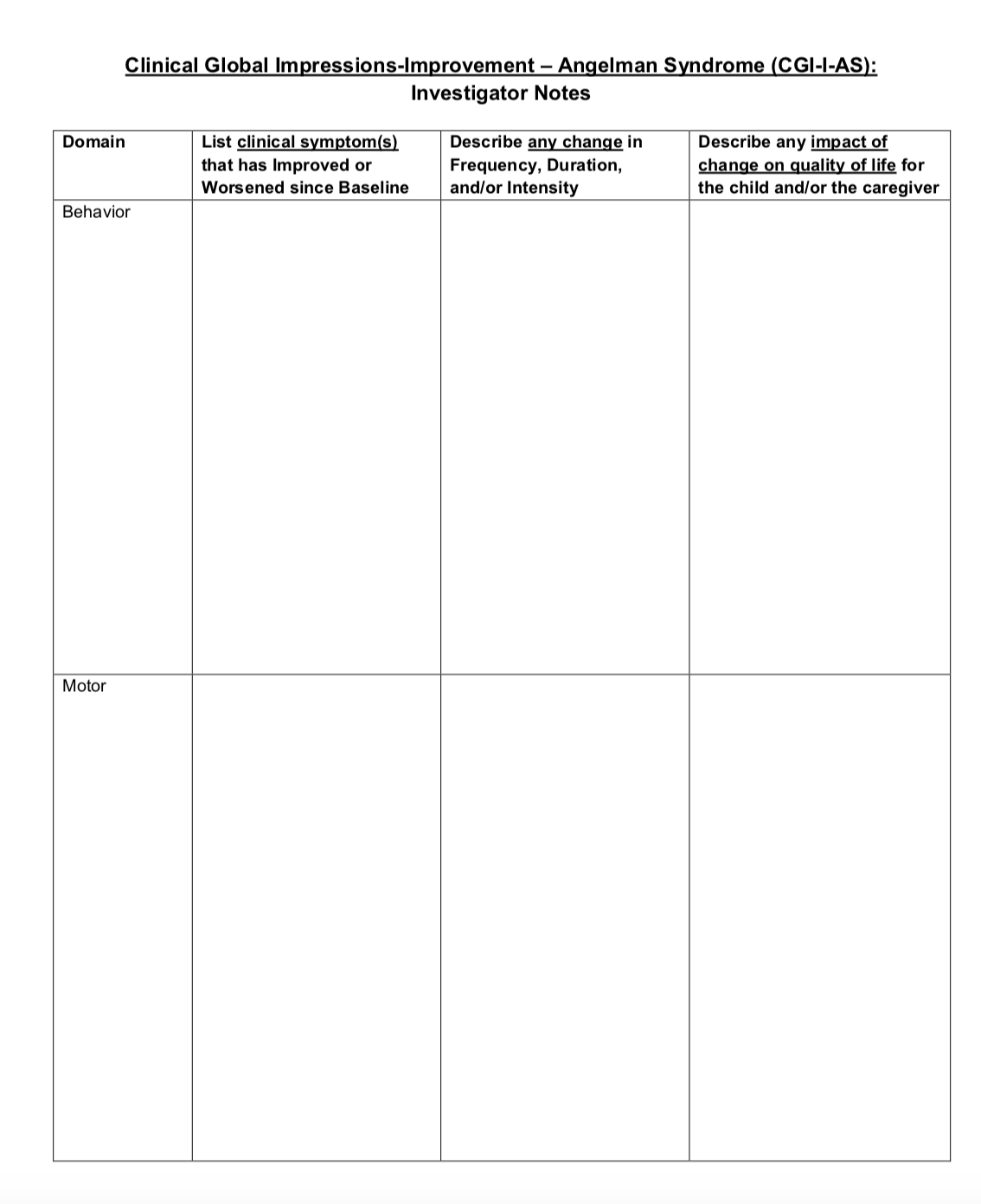

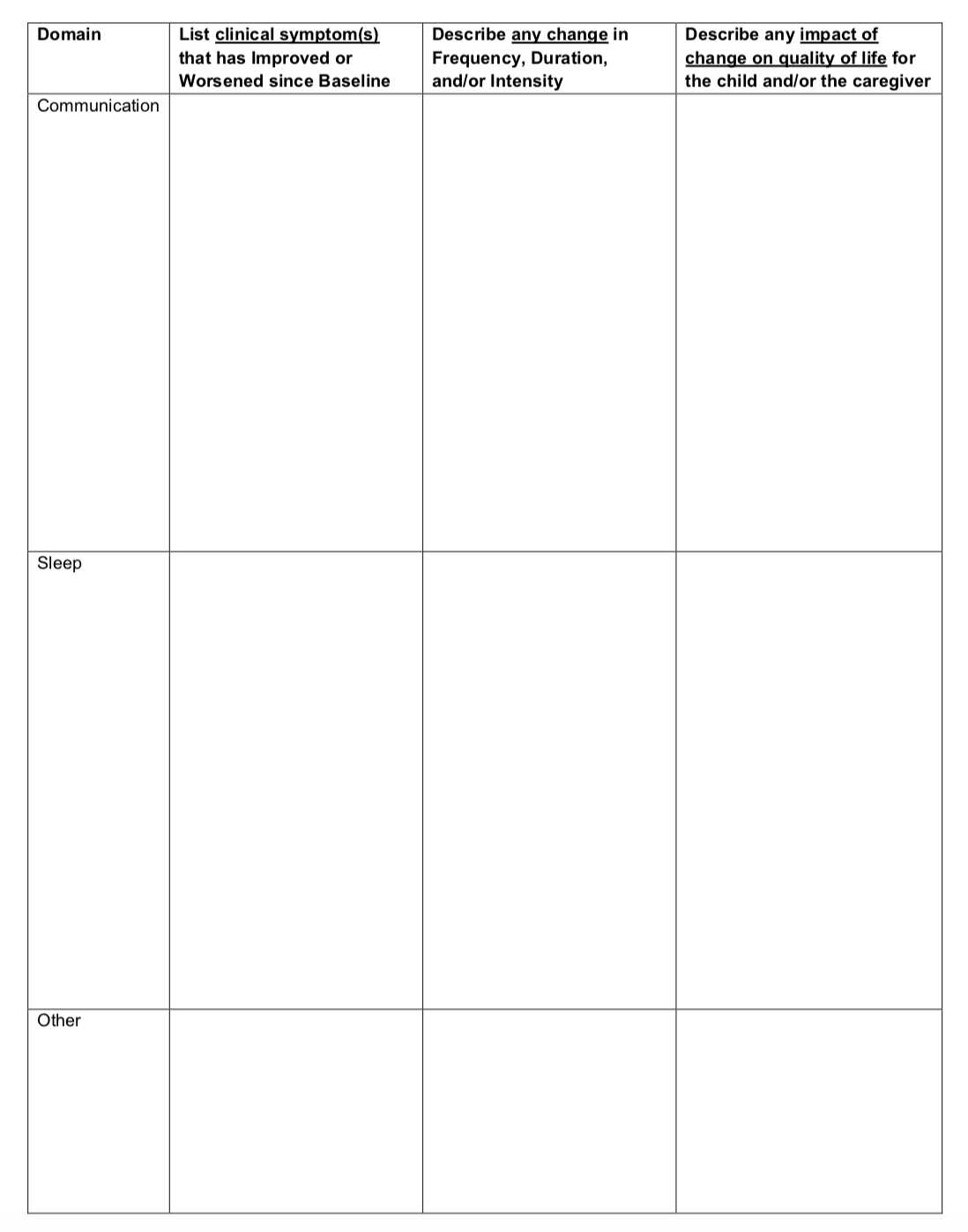

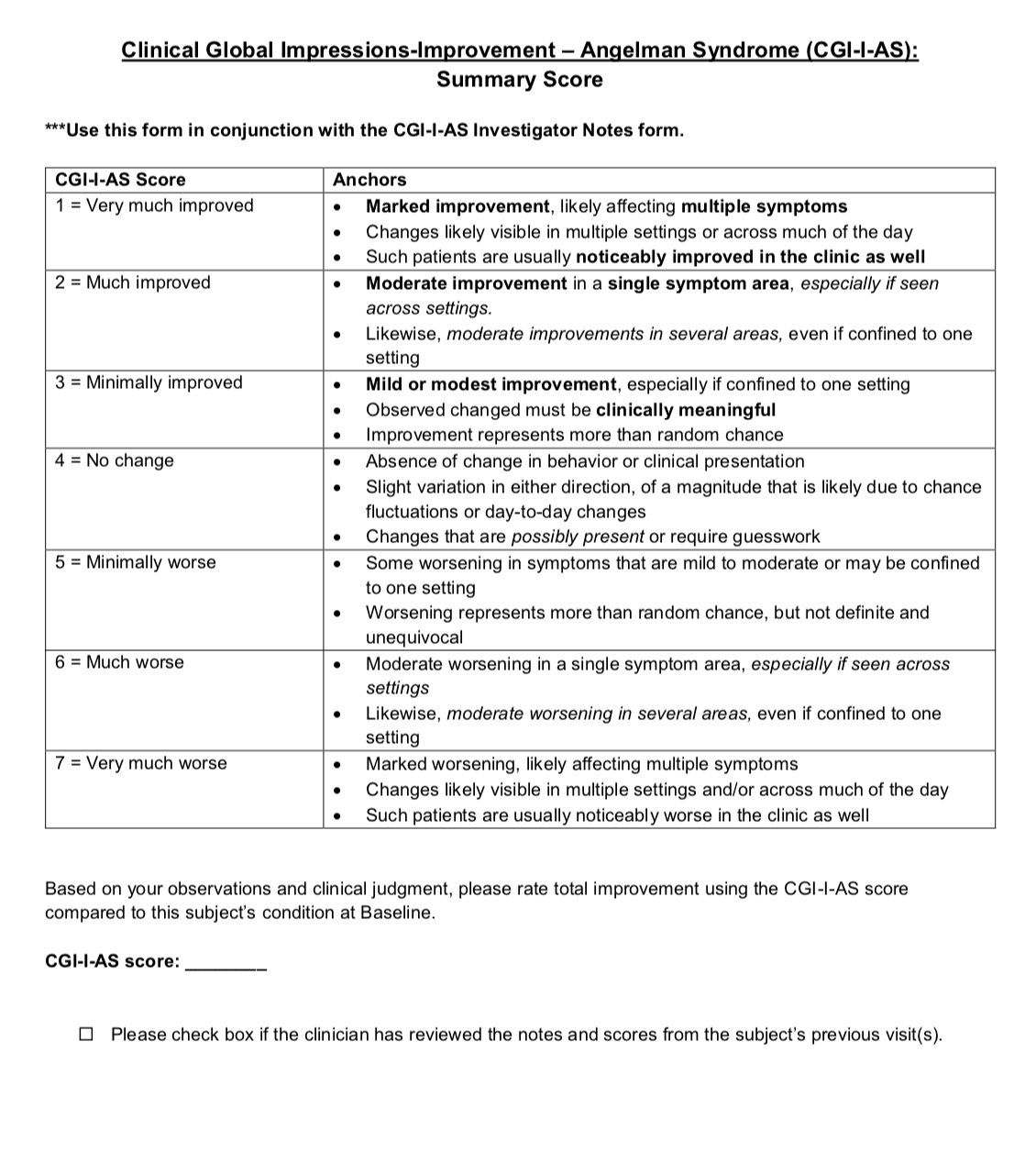
**
